# Identification of radiation‐induced EndMT inhibitors through cell‐based phenomic screening

**DOI:** 10.1002/2211-5463.12552

**Published:** 2018-12-06

**Authors:** Yeonhwa Song, Su‐Yeon Lee, A‐Ram Kim, Sanghwa Kim, Jinyeong Heo, David Shum, Se‐Hyuk Kim, Inhee Choi, Yoon‐Jin Lee, Haeng Ran Seo

**Affiliations:** ^1^ Cancer Biology Laboratory Institut Pasteur Korea Seongnam‐si Korea; ^2^ Assay Development and Screening Institut Pasteur Korea Seongnam‐si Korea; ^3^ Medicinal Chemistry Institut Pasteur Korea Seongnam‐si Korea; ^4^ Division of Radiation Effects Korea Institute of Radiological and Medical Sciences Seoul Korea

**Keywords:** CHIR‐99021, endothelial‐to‐mesenchymal transition, ionizing radiation, nonsmall cell lung cancer, radiation‐induced pulmonary fibrosis, visual phenomic screening

## Abstract

Radiation‐induced pulmonary fibrosis (RIPF) triggers physiological abnormalities. Endothelial‐to‐mesenchymal transition (EndMT) is the phenotypic conversion of endothelial cells to fibroblast‐like cells and is involved in RIPF. In this study, we established a phenomic screening platform to measure radiation‐induced stress fibers and optimized the conditions for high‐throughput screening using human umbilical vein endothelial cells (HUVECs) to develop compounds targeting RIPF. The results of screening indicated that CHIR‐99021 reduced radiation‐induced fibrosis, as evidenced by an enlargement of cell size and increases in actin stress fibers and α‐smooth muscle actin expression. These effects were elicited without inducing serious toxicity in HUVECs, and the cytotoxic effect of ionizing radiation (IR) in nonsmall cell lung cancer was also enhanced. These results demonstrate that CHIR‐99021 enhanced the effects of IR therapy by suppressing radiation‐induced EndMT in lung cancer.

**Abbreviations**
α‐SMA: alpha‐smooth muscle actin
ECM: extracellular matrix
ECs: endothelial cells
EGFR: epidermal growth factor receptor
EMT: epithelial–mesenchymal transition
EndMT: endothelial‐to‐mesenchymal transition
HCAECs: human coronary artery endothelial cells
HCS: high‐content screening
HUVECs: Human umbilical vein endothelial cells
IR: ionizing radiation
NSCLC: nonsmall cell lung cancer
RIPF: Radiation‐induced pulmonary fibrosis
TME: tumor microenvironment

Despite decades of research, systemic therapies fail to cure most lung cancers which ranks highest in terms of both incidence and mortality in the world 1, 2. There are two major histological types of lung cancer: small‐cell lung cancer and nonsmall cell lung cancer (NSCLC; i.e., adenocarcinoma, squamous cell carcinoma, large cell carcinoma) 3. NSCLC comprises 85% of lung cancer cases, and about 40% are unresectable 4, 5. Although radiotherapy is often used for patients with unresectable NSCLC tumors, it remains largely palliative due to radioresistance, which is most likely due to occur via tumor heterogeneity in terms of cell of origin, pathology, etiology, and molecular/genetic pathogenesis 6, 7, 8. Hence, researchers have aimed to elucidate target genes and drug candidates for overcoming radioresistance. Much to our dismay, the development of targeted drugs has not yet significantly improved outcomes.

Recent shift in the paradigm of radiobiology for cancer therapy has changed the researches from tumor cell genetics alone to the complicated crosstalk between cancer and the tumor microenvironment (TME) 9, 10, 11, 12. The cellular environment in which the tumor exists, including the surrounding blood vessels, immune cells, fibroblasts, other cells, signaling molecules, and the extracellular matrix (ECM), constitutes the TME. The endothelial cells (ECs) and the tumor vasculature are the most frequently studied components within the TME with respect to radiation 9, 13, 14, 15.

The structure of the vascular endothelium is composed of ECs, smooth muscle cells, and a basement membrane. ECs form a continuous and uniform monolayer in normal tissues and express various receptors of angiogenic factors, including vascular endothelial growth factor receptors, Tie‐2, epidermal growth factor receptor (EGFR), platelet‐derived growth factor receptor, and chemokine receptors 16, 17, 18, 19. Several signaling cascades to regulate survival, proliferation, and invasion are triggered by the activation of receptors in ECs. The EC has come to light as an additional source of fibroblasts during endothelial‐to‐mesenchymal transition (EndMT). EndMT is a phenotypic conversion of ECs to fibroblast‐like cells and is involved in cardiac development and tissue fibrosis 20. Particularly, in the TME, EndMT generates carcinoma‐associated fibroblasts and may be essential for cancer progression.

In general, the phenotypic screening approach has been shown to be the most successful approach for first‐in‐class drugs 21. The benefits of such screens are that they lead to the identification of novel targets, novel compound mechanisms of action, and new pathways of therapeutic value. However, the process of determining the relevant target or targets of molecules identified by phenotypic screening is often slow or impossible 22, 23.

In this study, we applied drug repositioning strategy 24, 25, which is the application of known drugs and compounds to treat new indications, to the phenotypic screening of ionizing radiation (IR)‐induced EndMT inhibitors for the development of a preclinical radioprotector to elucidate the mechanism of compounds from phenotypic screening and to find new pathways of EndMT.

## Materials and methods

### Cell lines and culture conditions

Human umbilical vein ECs (HUVECs) and human coronary artery ECs (HCAECs) were purchased from Lonza (Basel, Switzerland), and H1299 cells (NSCLC cell line) were purchased from Korean Cell Line Bank (Seoul, Korea). Cells were incubated at 37 °C in a humidified atmosphere of 5% CO_2_. For maintenance, HUVECs and HCAECs were cultured in EGM‐2 medium (Lonza, CC‐3162) supplemented with 1× penicillin–streptomycin (P/S; Thermo Fisher Scientific, Waltham, MA, USA), and H1299 cells were cultured in RPMI1640 (Welgene, Daegu, Korea) supplemented with 1× P/S and 10% fetal bovine serum (Gibco, Gaithersburg, MD, USA). Cells were passaged at approximately 80% confluence with complete growth media.

### Irradiation

Using a ^137^Cs source, cells were irradiated with a single dose of gamma radiation (10 Gy) at room temperature.

### Phenomic screening and assay validation

Collagen type 1 (BD Collagen I, 354236; Thermo Fisher Scientific) was diluted in 70% ethanol, and then, the collagen solution (400 μg·mL^−1^) was dispensed into each 384‐well plate (6007550; PerkinElmer, Waltham, MA, USA). After a 1‐h incubation at room temperature, the wells were rinsed twice with Dulbecco's phosphate‐buffered saline. HUVECs were seeded at a density of 7 × 10^2^ cells per well onto collagen‐coated, 384‐well plates (781956; Greiner Bio‐one, Frickenhausen, Germany) and allowed to attach in complete growth media. Cells were then exposed to 10 Gy radiation at room temperature. After irradiation, the cells were incubated for an additional 3 h. The compounds were tested at a final concentration of 10 μm in 0.5% DMSO (v/v) using an automated liquid handling system (Hummingbird, Analytik Jena, Jena, Germany). After the 72‐h treatment, cells were fixed with 4% paraformaldehyde (w/v; PFA). The expression of actin filament was determined by incubation with 488‐Phalloidin (1 : 100, MOP‐A7466; Thermo Fisher Scientific) using direct immunofluorescence staining. The nuclei were stained using Hoechst 33342 (1 : 1000, MOP‐H3570; Thermo Fisher Scientific) at room temperature (R.T.). For assay validation, a control run was performed. The low control consisted of three 384‐well plates that contained irradiated cells, and the high control consisted of three 384‐well plates that contained nonirradiated cells. These plates were fixed and stained using the procedures described above.

### Cell survival assay

H1299 or HUVEC cells were seeded at a density of 2 × 10^3^ and 1 × 10^3^ cells per well, respectively, onto 384‐well plates (781091; Greiner Bio‐One) and allowed to attach in complete growth media for 16 h. After being treated depending on the experiment condition, cells were fixed with 4% PFA and stained with Hoechst 33342 at R.T. To analyze the number of nucleus, image acquisition was performed using operetta and in‐house software tool and harmony 3.5.1^®^ high‐content imaging system (harmony, PerkinElmer). After counting the number of nucleus, the curve were fitted with fold induction relative to control.

### Small molecule library

The library used for the screen combined 622 compounds obtained from Selleckchem (Houston, TX, USA). The Selleck library contains diverse anticancer and kinase inhibitors. The chemical library was plated onto 384‐well intermediate storage plates (Greiner Bio‐One) prior to use. The screen was performed with 10 μm as the final concentration, diluted in 0.5% DMSO (v/v).

### Image acquisition and analysis

For detecting and visualizing filamentous actin (F‐actin) and nuclei, images were collected using an automated high‐content imaging system with a 20× magnifying objective (operetta, PerkinElmer). The acquired images were analyzed using an in‐house software tool and harmony 3.5.1^®^ high‐content imaging (harmony, PerkinElmer) for segmentation of cells.

### Western blot analysis

Human umbilical vein endothelial cells and HCAECs were pretreated with 1 μm CHIR‐99021 and then irradiated with 10 Gy. After a 24‐h incubation, cells were solubilized in lysis buffer (3M, Maplewood, MN, USA) and boiled for 5 min. Equal amounts of protein (30 μg per well) were separated on 8% SDS/PAGE gels. After electrophoresis, the proteins were transferred onto a nitrocellulose membrane (Pall Corporation, Port Washington, NY, USA) and blocked with 5% skim milk for 30 min at room temperature. After blocking, the membrane was incubated with rabbit anti‐human α‐smooth muscle actin (α‐SMA; 1 : 3000, ab32575; Abcam, Cambridge, UK), or mouse anti‐human β‐actin (1 : 10 000, A5441; Sigma‐Aldrich, St. Louis, MO, USA) for 16 h at 4 °C. After washing, the blots were incubated with horseradish peroxidase‐conjugated secondary antibody (Cell Signaling Technology, Danvers, MA, USA) at a 1 : 10 000 dilution. Specific bands were visualized by enhanced chemiluminescence (Thermo Fisher Scientific) and recorded on X‐Omat AR films (Eastman Kodak Co., Rochester, NY, USA).

### Statistical analysis

All experiments were performed at least three times. The results are expressed as the mean ± standard deviation (SD). Statistical analysis was performed using the Student's *t*‐test.

## Results

### Establishment of the visual phenomic screening platform to measure radiation‐induced EndMT

Cellular phenotype‐based assays have emerged on the principle that many cellular targets are involved in the control of cellular morphologies. A target‐free, high‐content screening (HCS) approach can be used for drug discovery and basic research into disease mechanisms. In this study, we used this technology to screen for inhibitors of radiation‐induced EndMT in lung cancer.

Generally, radiation therapy upregulates fibroblast markers such as fibroblast‐specific protein 1 (FSP1) and α‐SMA via epithelial–mesenchymal transition (EMT) and EndMT. As a result, it induces morphological changes in cells in the TME. Therefore, we aimed to define the morphometric signature of EndMT associated with radiation‐induced vascular disease.

To define distinctive morphometric signatures before and after IR treatment, we focused on the reorganization of F‐actin after IR. F‐actin stress fibers have been shown to be significant regulators of cell adhesion and EC spreading and are more easily modulated by radiation.

**Figure 1:**
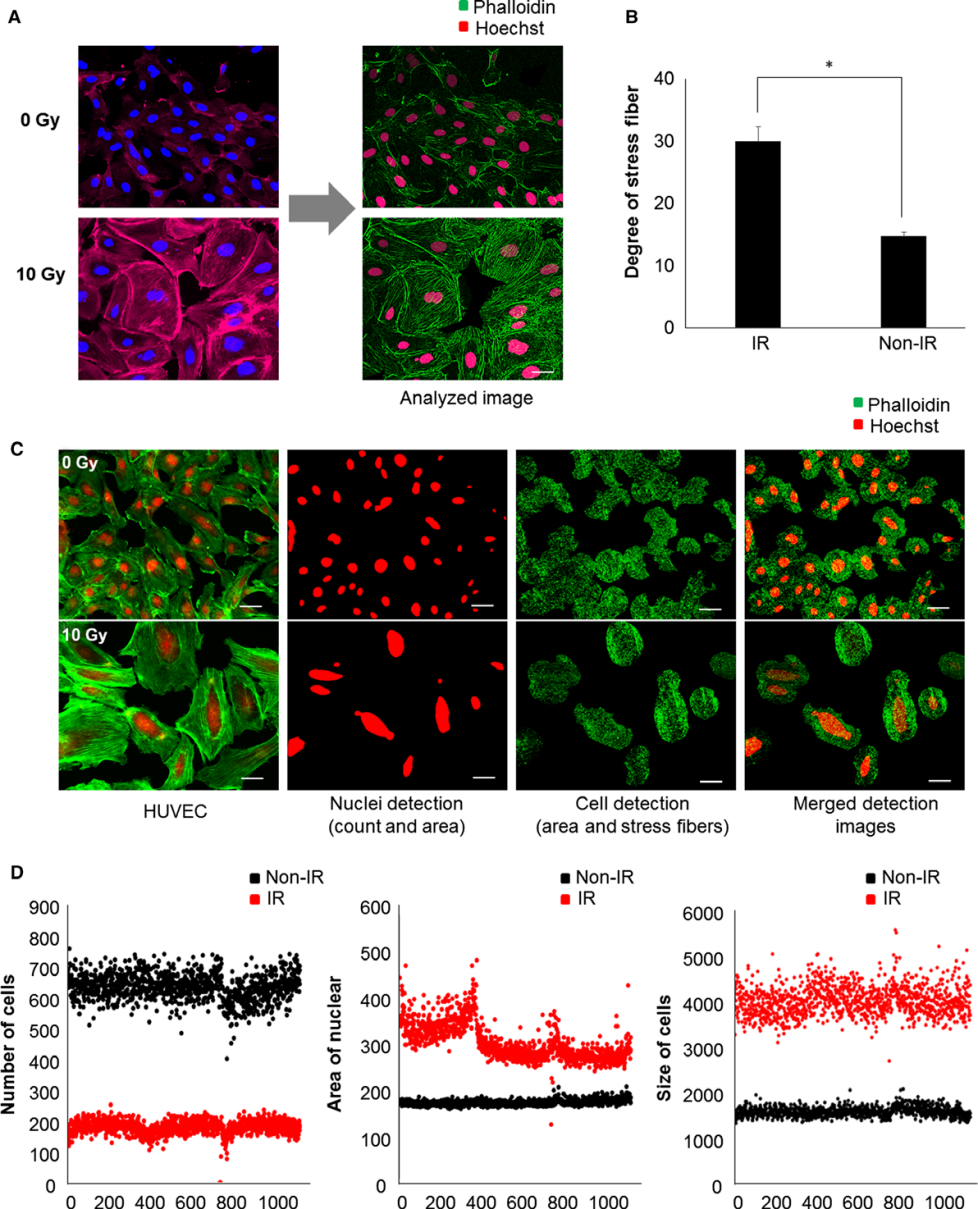
Radiation‐induced morphological changes. (A) HUVECs were treated with gamma radiation and stained with Phalloidin and Hoechst to assess morphological changes. Left side shows images acquired using the Operetta system, and the right side shows the segmentation algorithm applied by the im software (Institut Parsteur Korea, Gyeonggi‐do, Korea). (B) Quantification of the degree of stress fibers. Radiation stimulated the formation of actin microfilaments. Each error bar represents mean ± SD (*P < 0.0001). (C) Acquired images were analyzed using in‐house im software to detect the number of cells and area, as well as stress fibers. The segmentation algorithm determines nuclei count and size based on the intensity of objects above background. The actin filaments extend forward from the center of cell were measured to define distinctive morphometric signatures after irradiation. (D) High and low control plates were measured with several parameters to evaluate the assay in a 384‐well format. The distribution of each control showed the number of cells, nuclei, and cell area. Scale bar = 200 μm.

In HUVECs, IR exposure increased nuclei and cell size and decreased nucleus intensity. Staining of phalloidin, which contains fluorescent tags to visualize F‐actin, showed the formation of serially lined, thin stress fibers in the cytoplasm after IR treatment. F‐actin stress fiber bundles remained around the cell membrane (Fig. 1A). Based on these results, we developed an image analysis algorithm that can quantify the number of serially lined stress fibers following treatment with IR. IR led to a significant 1.7‐fold increase in the number of serially lined stress fibers in HUVECs (Fig. 1B). Next, to identify drugs minimizing radiation‐induced EndMT on HUVECs, 384‐well plates were set up for image‐based phenotypic screening. To confirm whether IR was evenly distributed in 384‐well plates, the cell number, nucleus area, and cell size were analyzed by harmony 3.5.1^®^ high‐content imaging and analysis software after treatment (Fig. 1C). IR induced a decrease in the number of HUVECs and increased nucleus area and cell size in all wells in an even manner (Fig. 1D).

These data suggest that serially lined F‐actin stress fibers are a proper morphometric signature of radiation‐induced EndMT. These findings were used to optimize the foundation of development in HCS technology that enables us to screen candidate drugs targeting regulators of radiation‐induced EndMT.

### Drug screening for regulators of radiation‐induced EndMT using the HCS system

**Figure 2:**
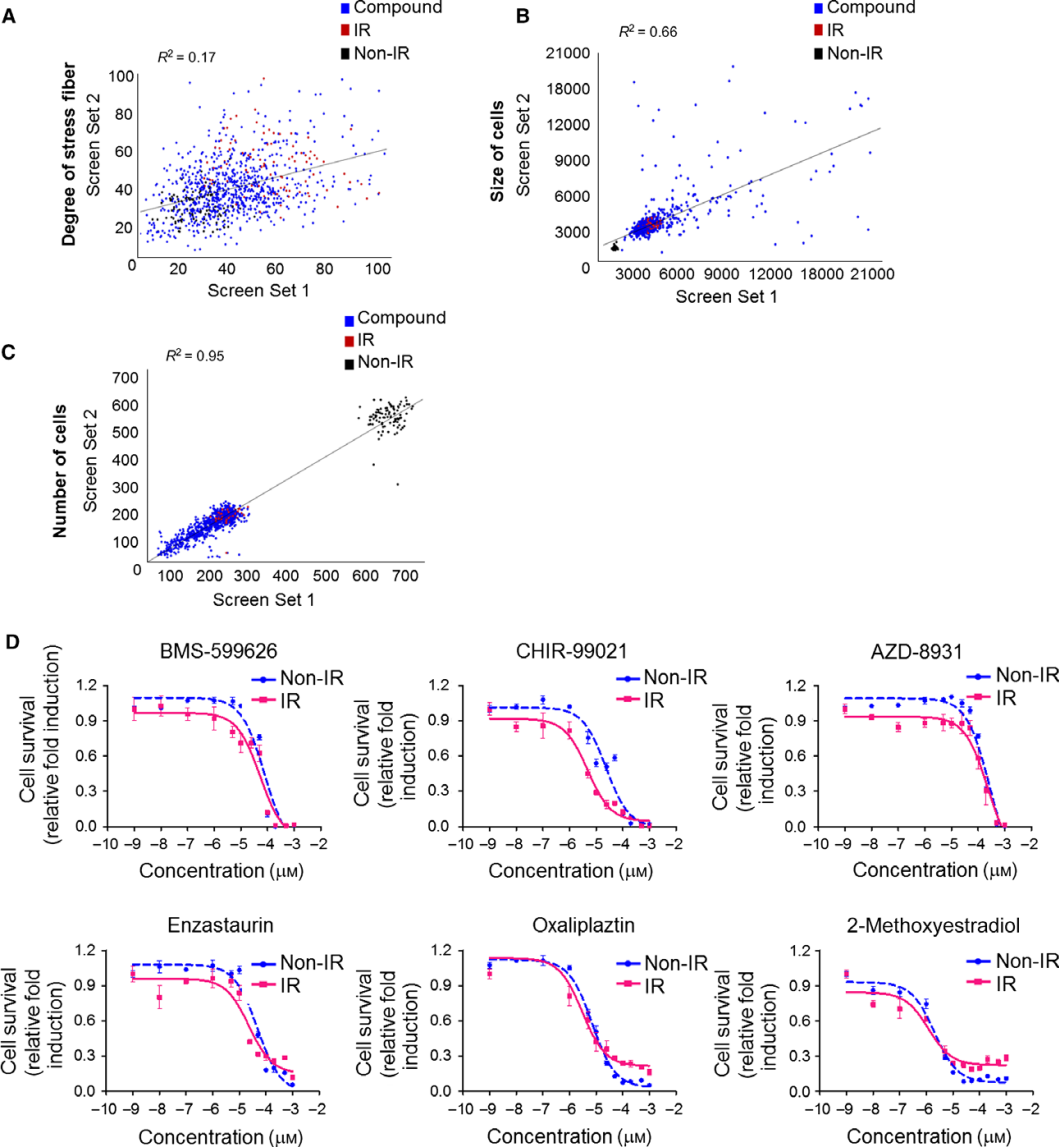
Phenomic screening results. Assay sensitivity was evaluated against a library containing 622 compounds screened in duplicate to assess reproducibility. (A) Scatter plot analysis shows the correlation of the degree of stress fiber in set 1 versus set 2. Each plot represents a single compound in the assay plate. (B) Scatter plot analysis of cell size and (C) cell number. (D) H1299 cells were irradiated with 10 Gy and treated with the six hit compounds at the indicated concentrations for 48 h at 3 h following irradiation. After incubation, cells were stained with Hoechst and analyzed with the Operetta system. Dose–response curve with cell survival of H1299 with or without radiation is shown.

Subsequently, we performed drug screening to identify compounds that specifically alter the degree of radiation‐induced serially lined F‐actin stress fibers without cytotoxicity in HUVECs. After 3 h of radiation treatment, compounds were screened at an initial concentration of 10 μm with a readout looking at the attenuation of IR‐induced stress fibers and enlargement of cell size (Fig. 2A,B), but not the number of cells (Fig. 2C). Positive and negative controls were 10 Gy IR and 0.01% DMSO, respectively.

Screening was then performed to identify compounds that block radiation‐induced EndMT in HUVECs. We used the Selleckchem Library, which is composed of 622 compounds, including the kinase inhibitor library and anticancer compounds library. Drugs were repositioned in duplicate to confirm the reproducibility of observed effects (Fig. 2A–C). We identified six positive compounds: BMS‐599626 [EGFR, human EGFR2 (HER2) inhibitor], CHIR‐99021 [glycogen synthase kinase (GSK)3β inhibitor], AZD‐8931 (EGFR, HER2 inhibitor), Enzastaurin (protein kinase C inhibitor), Oxaliplaztin (DNA/RNA synthesis inhibitor), and 2‐Methoxyestradiol (hypoxia‐inducible factor inhibitor).

Because our primary goal was to find the best compound that can improve anticancer effects in lung cancer by blocking radiation‐induced EndMT in ECs, we measured the growth inhibitory effects of the six HIT compounds on H1299 cells, which are NSCLC cells (Fig. 2D). The six HIT compounds inhibited cell growth in the following order: 2‐Methoxyestradiol > Oxaliplaztin > CHIR‐99021 > Enzastaurin > BMS‐599626 > AZD‐8931 (Table 1).

**Table 1:** Effect of HIT compounds on the growth of H1299 cells

Compounds	IG_50_ (μm)
BMS‐599626	78.12
CHIR‐99021	23.50
AZD‐8931	274.50
Enzastaurin	48.55
Oxaliplaztin	7.17
2‐Methoxyestradiol	2.05

To examine the radiosensitizing effects of the six HIT compounds, cell survival assays were performed with IR. Oxaliplaztin, 2‐Methoxyestradiol, and CHIR‐99021 exhibited sufficient radiosensitizing effects at concentrations below 5 μm (Fig. 2D, Table 2).

**Table 2:** Half maximal inhibitory concentrations (IC_50_) of HITs for radiosensitizing in H1299 cell lines

Compounds	IC_50_ (μm)
BMS‐599626	53.61
CHIR‐99021	4.81
AZD‐8931	236.50
Enzastaurin	23.22
Oxaliplaztin	3.05
2‐Methoxyestradiol	1.21

### CHIR‐99021 sufficiently attenuated radiation‐induced stress fibers in HUVECs

**Figure 3:**
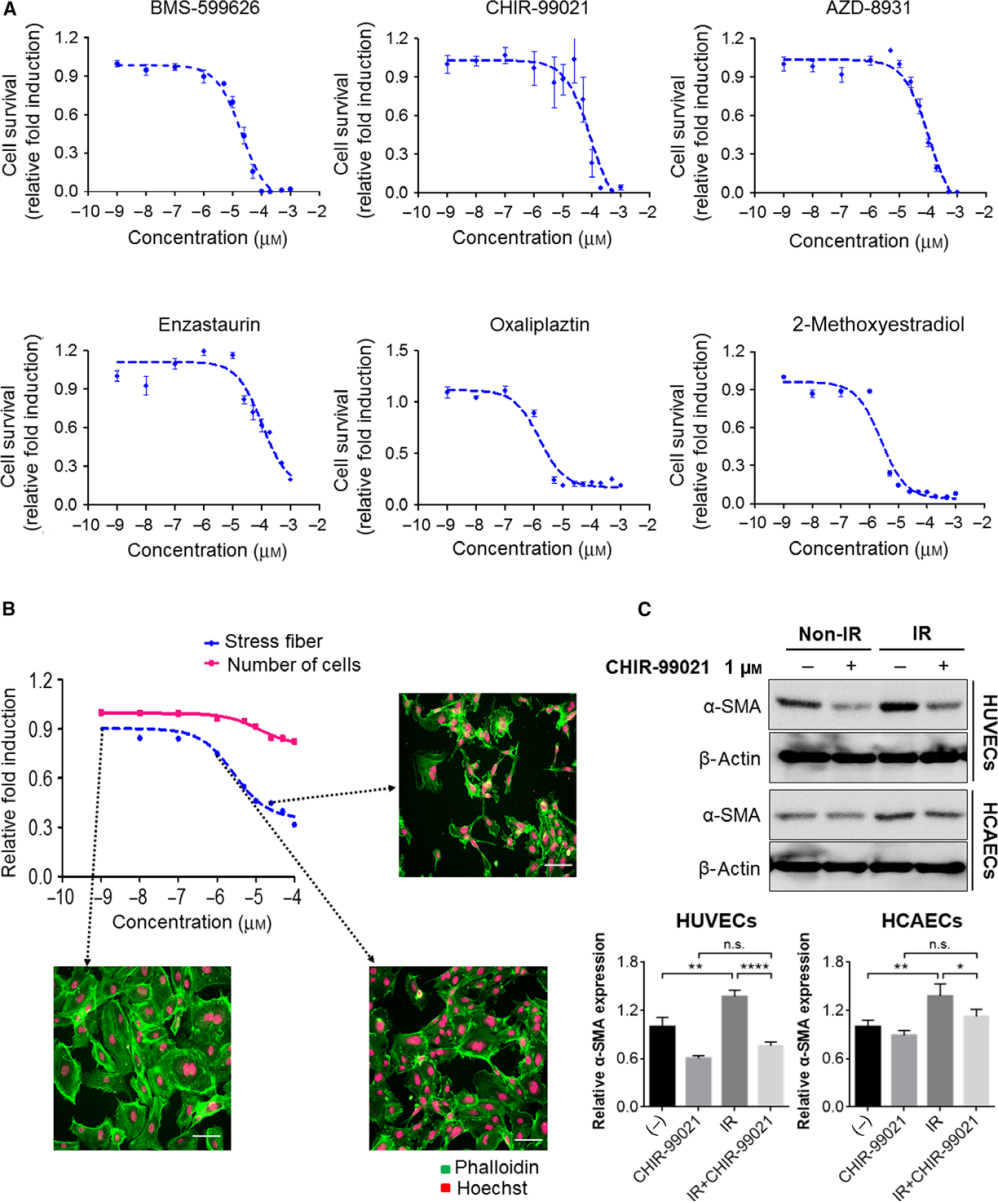
Effects of CHIR‐99021 on HUVECs. (A) HUVECs were treated with the six hit compounds at the indicated concentrations. After a 48‐h incubation, cells were stained with Hoechst, and cell numbers were analyzed with the Operetta system. (B) HUVECs were treated with CHIR‐99021, which was the final hit compound, at the indicated concentrations for 48 h. After incubation, cells were stained with Phalloidin and Hoechst. Number of cells and stress fibers were analyzed with im software. Each image shows stress fibers in HUVECs. Scale bar = 200 μm. (C) HUVECs and HCAECs were pretreated with CHIR‐99021 at 1 h before radiation and irradiated with 10 Gy. After 24 h, cells were prepared for western blot analysis. Expression of α‐SMA (EndMT‐related protein) was examined in HUVECs and HCAECs. β‐actin was loaded for control. The band intensity was quantified and calculated relative to Non‐IR/CHIR‐99021 0 μm group (lower). Statistical analysis was performed using the Student's *t*‐test and *n* = 3. Each error bar represents mean ± SD (*P < 0.05, **P < 0.01, ****P < 0.0001, ns: not significant).

Next, we measured the growth inhibitory effects of the six HIT compounds on HUVECs (Fig. 3A, Table 3). Comparison analysis of IG_50_ values between H1299 cells and HUVECs revealed that CHIR‐99021 could inhibit the formation of serially lined F‐actin stress fibers without cytotoxicity in HUVECs and have substantial radiosensitizing effects on NSCLC cells.

**Table 3:** Effect of HIT compounds on the growth of HUVEC cells

Compounds	IG_50_ (μm)
BMS‐599626	20.60
CHIR‐99021	87.88
AZD‐8931	106.1
Enzastaurin	105.5
Oxaliplaztin	1.522
2‐Methoxyestradiol	2.532

Dose–response studies were then performed to validate the potency of CHIR‐99021 on radiation‐induced EndMT in HUVECs. CHIR‐99021 has no effect on cell survival when administered with IR exposure in HUVECs. However, treatment with CHIR‐99021 downregulated the radiation‐induced formation of F‐actin stress fibers in a dose‐dependent manner (Fig. 3B). At 24 h after radiation treatment, the level of α‐SMA expression in HUVECs was significantly increased; however, radiation‐induced α‐SMA overexpression was significantly attenuated by pretreatment with 1 μm CHIR‐99021. In addition to HUVECs, another endothelial cell, HCAECs, also confirmed that radiation‐induced α‐SMA overexpression was reduced by treatment with CHIR‐99021 (Fig. 3C). These results suggest that CHIR‐99021 suppresses radiation‐induced EndMT and markedly increases chemo‐sensitivity in NSCLC cells.

## Discussion

In recent years, phenotypic approaches for small‐molecular‐weight compound screening assays have become more popular in drug discovery as an alternative strategy to target‐based approaches 21. This approach is particularly powerful because of its ability to target any protein and nonprotein cellular components involved in the selected phenotype. The goal of this study was to identify IR‐induced EndMT inhibitory compounds for the development of a preclinical radioprotector. We used the phenotypic screening approach (or PhenomicScreen at IPK), which uses sophisticated image‐mining tools to screen cellular disease models and cellular phenotypes for small molecules that change the observed phenotypes 26.

To construct prediction method of IR‐induced EndMT using the imaging *in vitro* without clinical trials, we observed the morphological change after IR treatment in HUVEC cells (Fig. 1). These morphological changes occur in response various stimuli, including drugs, radiation, UV, and food.

Generally, changes in the cytoskeleton and desmosomes might be important in the local anchorage of tumor cells and might have value for predicting the response of the tumor to radiotherapy 27.

In terms of EndMT or EMT, stress fibers are common in motile mesenchymal cells and are linked to the ECM via focal adhesions. These stress fibers are also significant regulators of cell adhesion and the spreading of ECs. Furthermore, they are more easily modulated by radiation 28, 29. Specifically, F‐actin stress fibers are essential to the development and maintenance of endothelial cell–cell junctional apposition and the structural integrity necessary for barrier function 30. The centrally located stress fiber bundles disappear in response to radiation, and it is this radiation‐induced reorganization in microfilament structure that mediates the response. In this study, we established phenotypic screening approaches for the discovery of IR‐induced EndMT inhibitors, based on the radiation‐induced reorganization of stress fibers (Fig. 1A). We optimized high‐throughput screening conditions with 10 Gy IR and observed a small but reproducible increase of 1.7‐fold in the formation of serially lined F‐actin stress fibers (Fig. 1B).

We then initiated the screening process with 622 compounds (Fig. 2A). The screening was performed in duplicate to ensure a better chance of selecting true‐positive hits. However, phenotypic screening approaches based on the radiation‐induced reorganization of stress fibers had a linear regression of *R* ^2^ = 0.27 in two replicate experiments that should be complementary for efficient large‐scale screening. The criteria for hits included cell area (< 3000 mm^2^), nuclei count (nontoxic > 100, toxic < 100), nuclei area (> 250 mm^2^), and degree of stress fibers (< 20). We selected 24 hits (or 0.03% of all samples) that were subsequently confirmed in full dose–response curves and identified 6 positive compounds: BMS‐599626, CHIR‐99021, AZD‐8931, Enzastaurin, Oxaliplaztin, and 2‐Methoxyestradiol. A set of specific secondary assays was developed to confirm the observed phenotypic changes, as well as to narrow down the list of hit candidates. Finally, we selected CHIR‐99021, a GSK3β inhibitor (Fig. 3B).

The most common cytokines that activate EndMT are the transforming growth factor‐β super family of proteins. Other signaling pathways have also been recently reported; these include Wnt/β‐catenin 31, Notch 32, HSPB1 33, and various receptor tyrosine kinases 34. GSK3‐β, a target of CHIR‐99021, is known to regulate tumor migration and invasion by controlling EMT. Signaling pathways that inactivate GSK3β, such as phosphatidylinositol 3 kinase/Akt and mitogen‐activated protein kinase, may promote the cell cycle, anti‐apoptosis, and invasion, thus facilitating tumor progression 35, 36, 37, 38. In terms of anticancer effects, inactivation of GSK3‐β by combined treatment with CHIR‐99021 reinforced the sensitivity to radiation.

Overall, we have identified serially lined F‐actin stress fibers as a phenomic marker of radiation‐induced EndMT. The morphometry of serially lined F‐actin stress fibers was applied to a visual phenomic screening platform to screen candidate drugs targeting regulators of radiation‐induced EndMT *in vitro*. This *in vitro* screening platform has the potential to be widely adopted in drug discovery research of EndMT‐related diseases. Based on the image‐based phenotypic analysis, we identified CHIR‐99021 and characterized its EndMT inhibitory activity. Our results suggest that the combined treatment of CHIR‐99021 and radiation may be a promising approach to overcome environment‐mediated drug resistance and for the treatment of EndMT‐related disorders in lung cancer.

## Author contributions

YS and Y‐JL designed the experiments, analyzed data, and prepared the manuscript. S‐YL, A‐RK, and SK performed cell culture and participated in the *in vitro* experiments. IC participated in design of the study as well as draft the manuscript. JH and DS carried out the HCS screening to identify inhibitor of radiation‐induced pulmonary fibrosis. HRS designed and was the overseer of the entire study.

## Conflict of interest

The authors declare no conflict of interest.
